# Spontaneous Spinal Epidural Hematoma Associated With Short-Term Dual Antiplatelet Therapy: A Case Report

**DOI:** 10.7759/cureus.29415

**Published:** 2022-09-21

**Authors:** Manar J Alahmadi, Kinan S Almolky, Djilali M Rezai

**Affiliations:** 1 Medicine, College of Medicine, Taibah University, Medina, SAU; 2 Neurosurgery, King Fahad Hospital, Medina, SAU

**Keywords:** antiplatelet therapy, quadriparesis, neck pain, stroke mimic, dual antiplatelet therapy, spinal epidural hematoma

## Abstract

Spinal epidural hematoma (SEDH), either spontaneous or traumatic, is a rare neurosurgical emergency. Typically, the natural history is a sudden onset of severe neck or back pain, associated with neurological deficit, either immediately or after a short period of the pain onset. MRI is the gold standard investigation. The mainstay of treatment is spinal decompression, in the form of laminectomy or hemilaminectomy, with the evacuation of the hematoma. The occurrence of SEDH has been strongly associated with coagulopathy, especially that induced by anticoagulant use. The association between SEDH and antiplatelet therapy has been scarcely reported in the literature. We report a case of spontaneous SEDH in a patient who was on dual antiplatelet therapy. Our case is unique because the patient had been using antiplatelet agents for only six weeks prior to this diagnosis. As antiplatelet agents are widely prescribed, physicians should be able to anticipate SEDH as a possible complication of dual antiplatelet therapy to facilitate early treatment and better outcomes.

## Introduction

Spinal epidural hematoma (SEDH) is a rare occurrence yet an important cause of acute and progressive neurological deficit that requires early diagnosis and management. It was first described in 1869 by Robert Jackson [1]. SEDH can be classified into two main categories: traumatic SEDH and spontaneous SEDH [2]. Traumatic SEDH can either result from direct trauma to the spine or iatrogenic procedures, such as lumbar punctures, epidural catheters, and spinal surgeries [3]. Spontaneous SEDH is defined by bleeding in the extradural space without an identifiable cause, such as preceding trauma, coagulopathy, and arteriovenous malformation [4]. According to the most recent evidence, the main risk factor for spontaneous SEDH is coagulopathy, either inherited or as a result of anticoagulant and antithrombotic agent use [5]. Other risk factors include atherosclerosis, hypertension, pregnancy, and deep diving [5].

Antithrombotic medications are nowadays widely prescribed. A rare yet critical association linked with their use is spontaneous SEDH [4,6-8]. Antithrombotic agents described in the literature to be implicated in spontaneous SEDH are aspirin, clopidogrel, and ticlopidine [8]. Here, we report a case of spontaneous SEDH in a female patient on short-term dual antiplatelet therapy with aspirin and clopidogrel. We aim to highlight a possible factor that might be implicated in the pathophysiology of this rare phenomenon to facilitate prompt recognition and treatment.

## Case presentation

A 67-year-old female with a history of smoking, diabetes mellitus type 2, hypertension, and ischemic heart disease presented to our emergency room with a two-hour history of sudden severe neck pain and stiffness, associated with weakness involving all extremities, but more prominent in the right upper extremity. There was no sphincter dysfunction. The patient denied any preceding trauma or a history of similar episodes. Six weeks earlier to this presentation, the patient suffered a myocardial infarction that was managed with percutaneous coronary intervention through the right femoral artery. The procedure was complicated by a pseudoaneurysm and puncture site hematoma that was surgically evacuated. Afterward, the patient was discharged home with aspirin 81 mg tab once daily, and clopidogrel 75 mg tab once daily. Her other home medications were perindopril 2.5 mg tab once daily, glimepiride 6 mg tab once daily, empagliflozin 25 mg tab once daily, and atorvastatin 20 mg tab once daily.

On physical examination, the patient’s vital signs were as follows: heart rate: 100 bpm; blood pressure: 160/80; arterial oxygen saturation (SaO_2_): 98% on room air; temperature: 37°C. Glasgow Coma Scale (GCS) score was 15/15. Neurological examination revealed generalized hyporeflexia and hypotonia, which were assumed to be the patient's baseline, and motor deficits involving all limbs. Power was assessed using the Medical Research Council motor grading scale. Power in the right upper limb was 0/5, in the left upper limb was 4/5, and in both lower limbs was 3/5. Babinski's sign was equivocal bilaterally. Sensation, including temperature, proprioception, and vibration, was intact, but it was subjectively reduced in the lower extremities compared to the upper extremities. The anal tone was preserved, and there was no saddle anesthesia. There were no cranial nerve deficits.

**Figure 1:**
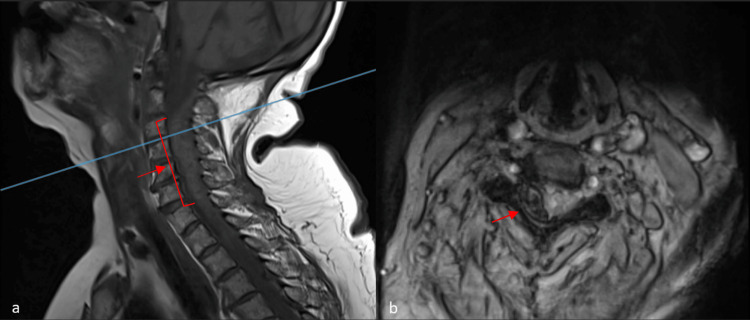
(A) T1-weighted MRI (sagittal view) showing a hyperintense collection from the C3 level down to the lower border of C7. (B) T2-weighted MRI (axial view) at the C3-4 level, where the hematoma is most prominent, showing complete obliteration of the exiting right nerve root.

Initially, a stroke code was activated by the emergency team, and an immediate computed tomography scan of the head and neck without contrast was carried out. It revealed right posterolateral extramedullary hyperdense oval-shaped mass, displacing and compressing the cord from C3-4 level down to C6-7. A cervical magnetic resonance imaging (MRI) was warranted, and it confirmed the diagnosis of cervical epidural hematoma, extending from C3 down to the lower border of C7 (Figure 1A). The axial view demonstrated a significant compression on the right nerve root (Figure 1B). Laboratory investigations, including the coagulation profile, were unremarkable. However, bleeding time was uninvestigated; hence, platelets dysfunction due to dual antiplatelet therapy cannot be presumed with complete certainty to be the main cause of bleeding.

**Figure 2:**
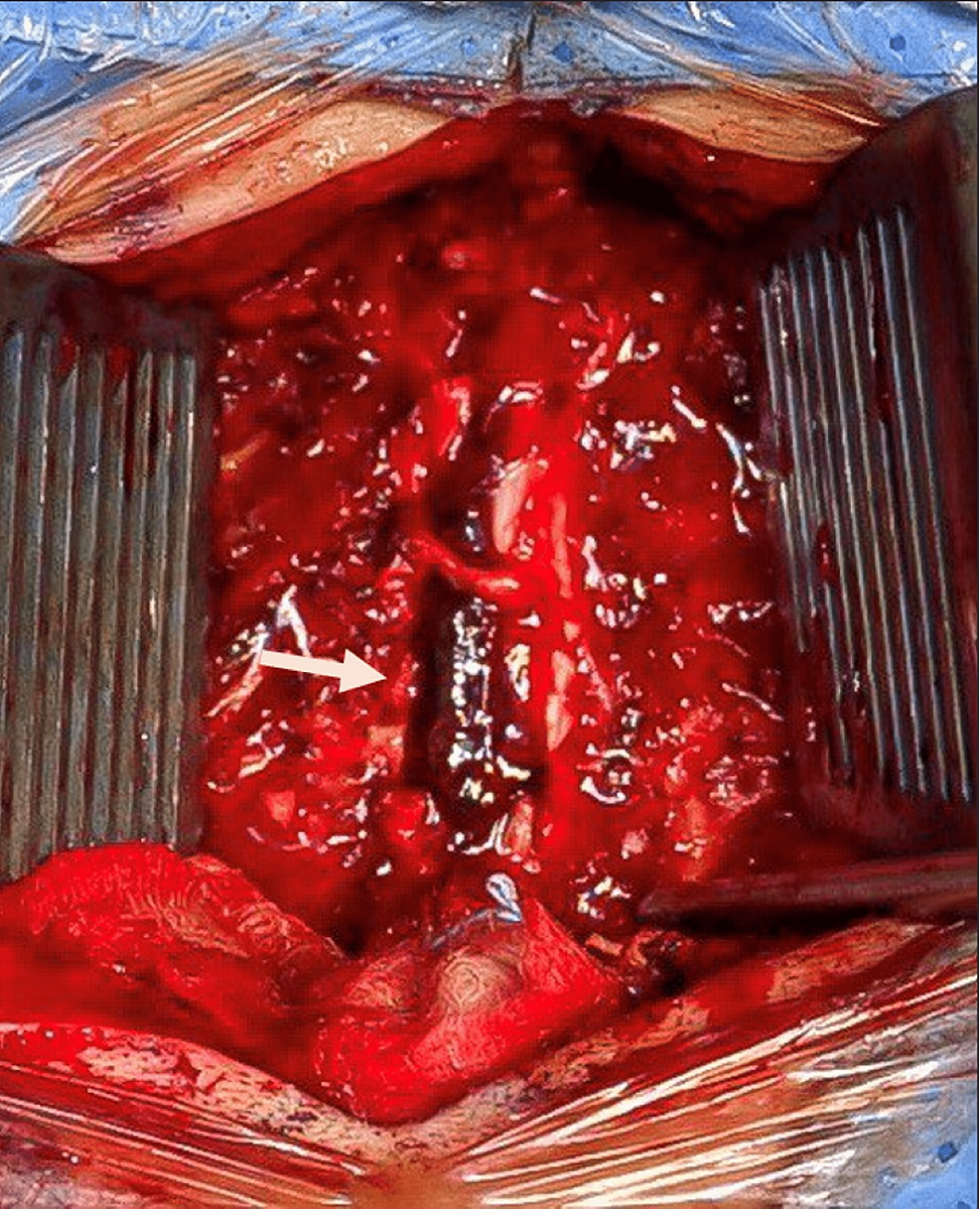
Gross intraoperative picture of the epidural hematoma.

Although the American College of Cardiology/American Heart Association (ACC/AHA) recommends waiting for at least four to six weeks before any surgical intervention after an attack of myocardial infarction [9], an urgent surgical decompression was mandatory for our patient to prevent further deterioration and the development of permanent neurologic deficit. Emergency spinal decompression was carried out, with a time gap of 12 hours between the onset of symptoms and the time of surgery. Laminectomy of C3-6 and hemilaminectomy of C7 with the evacuation of the epidural hematoma were performed. Figure 2 shows an intraoperative picture of the hematoma. No intraoperative complications were encountered, and a blood transfusion was not needed.

To date, there are no clear guidelines in regard to the most appropriate time to restart antiplatelet therapy after surgical intervention in cases of spontaneous SEDH [8]. In our case, we restarted aspirin and clopidogrel on the first postoperative day after consulting our cardiologist, who recommended obtaining an ECG and cardiac enzymes analysis every eight hours for 24 hours postoperatively. On the first postoperative day, the patient was shifted to the intensive care unit for close observation. She was conscious and vitally stable, with a GCS score of 15/15, still complaining of right scapular pain and monoplegia in the right upper extremity, with minor residual weakness in the other extremities. On postoperative day three, the patient started to have physiotherapy sessions, mainly stretching and strengthening exercises of all limbs. She was gradually regaining movement in her right upper limb, with the power grade being 2/5 proximally and 0/5 distally. The power in the left upper limb and both lower limbs was 5/5. On postoperative day 10, the patient was discharged home. At discharge, she was ambulatory and no longer complained of neck pain, but she had minimal weakness in her right upper limb.

**Figure 3:**
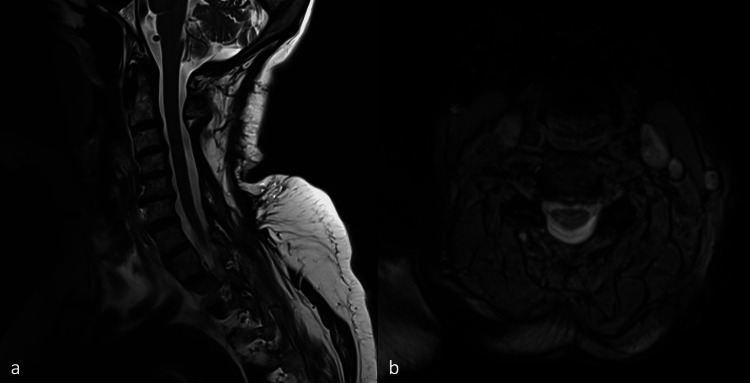
T2-weighted MRI of the cervical spine six months postoperatively. (A) Sagittal view. (B) Axial view at the C3-4 level.

After six months of follow-up, the patient was conscious, oriented, and ambulatory, with no recent complaints of neck pain; however, the patient still had residual weakness in her right upper extremity, with a power grade of 3/5. The power grade in her other extremities was 5/5. A follow-up MRI showed no recurrence of the hematoma (Figure 3).

## Discussion

SEDH is a rare entity, accounting for less than 1% of all spinal space-occupying lesions [10]. Spontaneous SEDH, which is defined as hemorrhage into the epidural space without a traumatic or iatrogenic etiology, has an incidence of 0.1 in 100,000 per year [10]. Affected patients are usually in their fourth to fifth decades of life [11,12]. The incidence is slightly increased in males compared to females (1.4:1) [10].

Risk factors underlying spontaneous SEDH are still poorly defined. Proposed risk factors include arteriovenous malformation, anticoagulant use, vertebral hemangioma, and hypertension [10]. In addition, minor trauma, pregnancy, hemophilia, and leukemia have been reported to be associated with spontaneous SEDH [12]. In a recent study, 14 patients diagnosed with spontaneous SEDH were thoroughly examined, and it was found that 71% of them received anticoagulation and/or antiplatelet therapy [5]. Nevertheless, up to 40-60% of patients when investigated appear to have none of these risk factors [10,11]. Unlike its higher prevalence with anticoagulants, only a few cases have been described in the literature linking spontaneous SEDH to antiplatelets [4,8]. In most cases, the first clinical manifestation is a sharp agonizing neck or back pain of a sudden onset [5,10]. This could be associated with a neurologic deficit, either immediately with the onset of pain or after a slight delay [10,12]. The onset of neurological deficit can range from hours, days, or even months from the onset of back pain [10,12]. The neurologic deficit typically takes the form of a lower motor neuron lesion, that is, hypotonia, hypo- or areflexia, and flaccid paralysis [5,10].

A clear anatomic background is vital to understanding the pathophysiology of SEDH. Like the brain, the spinal cord is covered by three meningeal layers (pia, arachnoid, and dura matters) from inside to out [13]. The dura and arachnoid matters are closely related, with only a potential space between the two, i.e., the subdural space [13]. Unlike the cranial subdural space, the spinal subdural space does not contain bridging veins; this might account for the fact that spinal epidural hemorrhage is more prevalent than spinal subdural hemorrhage [13]. The epidural space is the space between the dura mater and the vertebral column. It is divided by connective tissue, including plicae and meningovertebral ligaments into anterior, lateral, and posterior compartments [14]. Although the pathophysiology is not fully understood yet, it is thought that the source of bleeding in SEDH is the epidural venous plexus [10,15]. This theory is supported by the fact that this venous system is a low-pressure, valveless system, thus, it is susceptible to rupture when intra-abdominal or intrathoracic pressure is increased, such as during the Valsalva maneuver [10,12,15]. Other sources of bleeding include epidural arteries and arteriovenous malformation [10,12]. A rapidly evolving neurologic deficit would suggest an arterial source of bleeding rather than a venous source [11,15]. The dorsal extradural space is affected more often than the ventral extradural space [5,10-12]. This could be the result of two anatomical facts; one being that the ventral extradural plexus is partially supported by the posterior longitudinal ligament, and the other being that the dorsal extradural plexus is larger than the ventral extradural plexus [10].

The diagnosis of SEDH is challenging and requires a high index of suspicion, as there is a wide range of differential diagnoses, including cerebrovascular accident, disc herniation, aortic dissection, and subarachnoid hemorrhage [6]. For the diagnosis of SEDH, MRI is the gold standard investigation [10,12]. The features of SEDH differ over time, depending on its stage [10,13]. Table 1 demonstrates the MRI features of SEDH [13]. If there is active hemorrhage into the hematoma, an area of central enhancement would be visible on MRI with contrast [10].

**Table 1:** Radiologic features of spinal hematoma on MRI over time.

Stage	Time	T1	T2
Hyperacute	<24 hours	Hypointense	Hyperintense
Acute	1-3 days	Isointense	Hypointense
Subacute (early)	1-7 days	Hyperintense	Hypointense
Subacute (late)	1-2 weeks	Hyperintense	Hyperintense
Chronic	>2 weeks	Hypointense	Hypointense

Management guidelines are still vague. Early surgical decompression has been correlated with better improvement when compared to conservative management or delayed surgical intervention [4,10,11]. Delay in surgical intervention has been associated with poor outcomes [8,15]. It has been suggested by many that for optimal outcomes, surgical decompression should be performed within 24-36 hours of the onset of complete deficit, and within 48 hours for incomplete deficit [10,12]. The pathophysiology underlying the neurological deficit is not only due to the direct compression by the hematoma but is also a result of an inflammatory reaction [16]. Thus, prompt surgical intervention is indicated even in cases where there is a complete deficit [10]. The recovery of some patients who presented with a complete deficit (American Spinal Injury Association (ASIA) stage A) and demonstrated a full recovery (ASIA stage E) following surgical intervention had been reported in the literature [17]. The surgery of choice is usually either a laminectomy or a hemilaminectomy [10]. In a relevant study, complete laminectomy was preferred over hemilaminectomy, as it permits complete evacuation of the hematoma, and allows for adequate decompression [15]. Although a few cases of SEDH have been reported to resolve with conservative management, it is advised that this approach be adopted only in cases where rapid neurologic improvement is seen, and in patients who are not surgical candidates or are asymptomatic [4,10,15].

The prognosis of SEDH is dependent on many factors. In a literature review published in 2013, a total of 1177 cases of SEDH were reviewed to determine factors influencing outcomes after surgical decompression [2]. It was concluded that factors influencing outcome are (1) patient age and sex, (2) cause of the hematoma (spontaneous or traumatic), (3) site of the hematoma, (4) degree of neurological deficit at the time of treatment, (5) speed of onset of the neurological deficit, (6) type of treatment, and (7) postoperative rehabilitation. Among these factors, the most important indicators are the preoperative neurologic status, the speed of progression of the neurologic deficit, and the timing of surgical decompression [2]. In another review, it was stated that the most important factor is the preoperative neurologic status [10]. Factors that suggest a worse prognosis are a rapid progression of neurologic deterioration, the large size of the hematoma (involvement of ≥4 spinal segments), and a lack of sensory sparing [10].

## Conclusions

Although rare, the disastrous outcomes of untreated SEDH prompt the need for early recognition and treatment. With the increased use of antiplatelet therapy in the context of cerebrovascular accidents and ischemic heart disease, physicians should be aware of spontaneous SEDH as a possible complication of dual antiplatelet therapy. This would facilitate early treatment and, as a result, better outcomes.
